# Specialisation of Yeast Genera in Different Phases of Bee Bread Maturation

**DOI:** 10.3390/microorganisms8111789

**Published:** 2020-11-14

**Authors:** Roxane Detry, Noa Simon-Delso, Etienne Bruneau, Heide-Marie Daniel

**Affiliations:** 1 Laboratory of Mycology, Earth and Life Institute, Applied Microbiology, Université Catholique de Louvain, 1348 Louvain-la-Neuve, Belgium; detry.roxane@gmail.com; 2 Beekeeping Research and Information Centre (CARI), 1348 Louvain-la-Neuve, Belgium; simon@cari.be (N.S.-D.); bruneau@cari.be (E.B.); 3 Belgian Coordinated Collection of Microorganisms (BCCM), Mycothèque de l’Université Catholique de Louvain (MUCL), 1348 Louvain-la-Neuve, Belgium

**Keywords:** *Apis mellifera*, bee bread, diversity, ecology, microbiota, pollen, yeasts

## Abstract

Pollen stored by bees undergoes a fermentation marked by the presence of lactic acid bacteria and yeasts. It results in bee bread. Past studies have singled out *Starmerella* (*Candida*) *magnoliae* as the most common yeast species in honey bee-stored bee bread. *Starmerella* species are ecological specialists with potential biotechnological value. The rarity of recent studies on yeasts in honey bees prompted us to generate new information on yeast diversity during the conversion of bee-collected pollen to bee bread. Bees and stored pollen from two apiaries in Belgium were sampled, a yeast isolation protocol was developed, yeast isolates were grouped according to their macro- and micromorphology, and representative isolates were identified using DNA sequences. Most of the 252 identified isolates belonged to the genera *Starmerella*, *Metschnikowia*, and *Zygosaccharomyces*. The high abundance of yeasts in fresh bee bread decreased rapidly with the storage duration. *Starmerella* species dominated fresh bee bread, while mostly *Zygosaccharomyces* members were isolated from aged bee bread. *Starmerella* (*Candida*) *apis*, a rarely isolated species, was the most frequent and abundant species in fresh bee bread. Yeasts from the bee’s honey stomach and from pollen pellets obtained from bees hind legs were dominated by *Metschnikowia* species. The distinctive communities from pollen pellets over fresh bee bread to aged bee bread indicate a non-random distribution of these yeasts.

## 1. Introduction

Honey bees (*Apis mellifera*) not only collect and store nectar, but also pollen. Nectar and honey serve as their main carbohydrate source, while pollen serves as a source of proteins, lipids, vitamins and minerals [1]. Most of the pollen is consumed by nurse bees who rely on it for the production and secretion of amino acid-rich royal jelly. The jelly, and later a jelly/pollen mix, is fed to the worker and drone larvae until they seal themselves in their cocoons. The queen larvae receive a steady diet of royal jelly throughout their development. Appropriate nutrition induces the development of the flight muscles, the hypopharyngal glands and the ovaries [2].

To collect and store pollen in the hive, the floral pollen is moistened by forager bees with salivary secretions containing small amounts of their honey stomach content and loaded into receptacles on their hind legs called corbiculae [3]. In the hive, the foragers deposit the pollen pellets from their corbiculae in wax comb cells to be conditioned by workers. During this conditioning, workers add more secretions that are assumed to contain beneficial microorganisms, enzymes, and honey. Pollen stored by bees undergoes a mixed lactic acid fermentation marked by the presence of lactic acid bacteria and yeasts. This fermentation results in bee bread. During the brood-rearing season, the bee bread is stored for only a few days, while excess bee bread may be stored for several months. In certain circumstances, but not typically, bee bread cells may be capped by wax, propolis, or a mixture thereof [4,5].

The chemical composition of floral pollen is highly variable depending on the plant species with amounts of up to 22% starch, 20% lipids, considerable quantities of water-soluble vitamins, and usually less than 20% water [6]. Bees have been observed to collect pollen with 12–61% protein, but not pollen that is unusually rich in protein [7]. Bee bread differs from fresh pollen most notably by the degradation of starch, a lowered pH from 4.7 to around 4, and higher concentrations in amino acids [8,9,10]. There is controversy whether bee bread has a higher nutritive value compared to fresh pollen [11,12], or not [9,13,14]. The preference of bees for fresh bee bread (1–4 days) over longer-stored bee bread has been interpreted as contradictory to a higher nutritional value of bee bread [13,14]. In addition, the consumption of 21-day-old compared to 14-day-old bee bread had a negative impact on colony health and gut microbiome [15]. Agreement seems to exist on the preservative effect of pollen through its storage by bees [13,16]. Antibacterial activity was more pronounced in the top pollen and honey layer of bee bread cells compared to the bottom layer with the highest content in phenolic compounds [4].

The microbial activity in bee-collected pollen has been evidenced by viable cell counts of maximal 10^5^ bacterial colony-forming units (CFU) per gram of bee bread [13], which is lower compared to other spontaneously occurring mixed lactic acid–yeast fermentations such as in cocoa processing, with 10^8^ to 10^9^ CFU/g [17], likely because of the low water activity in pollen. The microbial succession in honey-bee-collected pollen reported by [18] showed maximum yeast counts of 12 CFU/g at 24 h and 48 h and a lactic acid bacteria peak at 48 h in the same magnitude as reported by [13], eventually terminating in a microbially inactive pollen. Yeasts have been observed in bee bread in far lower cell densities than bacteria and have shown a peak during the first week of storage [18,19]. Only one species, *Starmerella* (*Candida*) *magnoliae*, occurred consistently in pollen pellets and bee bread of up to 6 weeks in age, but not in floral pollen, suggesting that bees may have added this species to the pollen and the conditions during pollen storage selected for its survival [19].

The assumption of beneficial microbiota in bee-stored pollen rests largely on the recognition of specific bacterial core microbiomes in bees and bee bread, also present in the floral environment [20,21,22]. There is a lack of recent studies that address yeasts in honey bee-collected pollen and bee bread [23]. Next-generation sequencing studies of fungal bee microbiota use short sequences that result in genus-level identification but do not allow reliable species-level identification (e.g., [24,25]). However, a yeast-species-dependent effect on bee fitness was recognised by the addition of different yeast species to artificial nectar fed to bumble bees [26]. Physiologically and ecologically different groups of yeasts may also be involved in pollen resources of honey bees with potentially different effects on bee fitness, and it seems appropriate to address the yeast diversity during the storage and maturation of bee bread by culture-based techniques. The aim of the presented study was to improve the understanding of the yeast species involved in the conversion of bee-collected pollen to bee bread.

## 2. Materials and Methods

### 2.1. Samples

**Figure 1:**
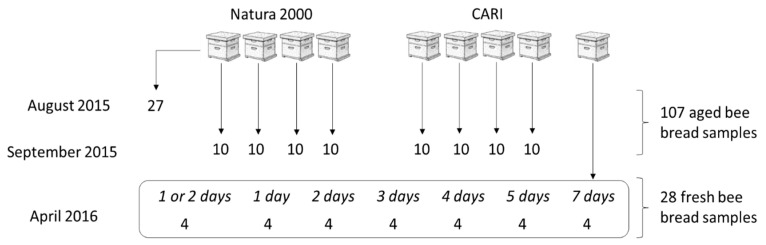
Numbers of pollen samples obtained from two apiaries located in Belgium in a Natura 2000 site, Chaumont-Gistoux and in a mixed zone with agriculture and small industry (CARI) in Louvain-la-Neuve. Samples originated from nine hives and are distinguished into aged (>20 days) and fresh bee bread (≤7 days).

Two apiaries in Belgium were sampled: a private apiary located in a Natura 2000 zone (sitecode: BE31007C0, sitename: Vallée du Train, https://natura2000.eea.europa.eu) in Chaumont-Gistoux (50.692770, 4.698056) and a research apiary of the Beekeeping Research and Information Centre (CARI), located in a mixed zone with agriculture and small industry in Louvain-la-Neuve (50.662900, 4.624573). Bee bread samples were obtained on three occasions (Figure 1). Samples of 27 pollen-filled cells from one honeycomb frame of the Natura 2000 apiary served to establish the sampling and cultivation methods in August 2015. Ten samples per hive were obtained from four healthy hives in both apiaries in September 2015, and these 80 samples were kept at room temperature for about 20 days to represent aged bee bread. A second series of 28 fresh bee bread samples were obtained from the CARI apiary in April 2016. Two wax foundation frames were introduced into a healthy hive and surveyed daily for pollen-filled cells, which occurred on the second day. The frames were then taken out, and then four pollen-filled cells, considered as day one, were sampled and the frames were stored aseptically at 30 °C to imitate the hive temperature. More sets of four samples were obtained daily until day seven. Samples of days one to three were taken from frame one, and the remaining samples from frame two. Bee bread samples were obtained using a sterile plastic pipette tip, cut to an opening of 3–4 mm, which was pressed into the pollen filling of a wax cell. The majority of the cell’s pollen content was transferred with the tip to a tube, weighed, and suspended in 600 or 750 mL saline.

Pollen pellets from foragers were sampled as the raw material to build up in-hive pollen stores. Honey stomachs were sampled because small amounts of their content are added to floral pollen by forager bees to attach the pollen as pellets to their hind legs [3]. In October 2015, fourteen forager bees from the CARI apiary were collected in one day without determining to which colony they belonged. The forager bees were frozen until dissection of their honey stomachs. Dissections according to the procedure for the rapid removal of the alimentary canal [27] were performed aseptically. Pollen pellets attached to the hind legs of 12 of these bees were used for yeast cultivation. Dissected honey stomachs and pollen pellets were suspended in 200 µL saline.

### 2.2. Cultures

**Figure 2:**
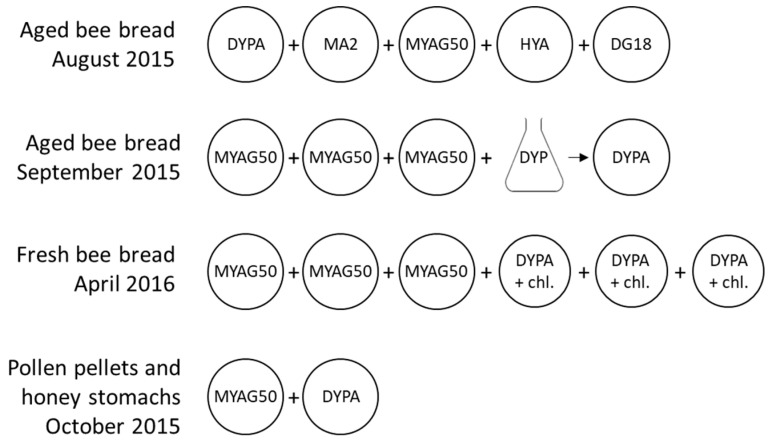
Overview of cultures on Dextrose Yeast Extract Peptone Agar (DYPA), Malt Agar 2% (MA2), Malt Yeast Agar Glucose 50% (MYAG50), Honey Yeast Agar (HYA), Dichloran-Glycerol Agar (DG18), and DYPA with chloramphenicol 200 mgL^−1^ (DYPA+chl). Single plating was performed to determine the most appropriate media on 27 bee bread samples and on pollen pellets and honey stomachs because of the small sample quantities. Triplicate plating was performed on 80 bee bread samples.

Five cultivation media (Table 1, Figure 2) were tested on 27 pollen samples collected in August 2015. Based on the observed growth, Malt Yeast Agar Glucose 50% (MYAG50) was selected for osmophilic yeasts and Dextrose Yeast Extract Peptone Agar (DYPA) for general yeast growth. Aged bee bread samples (September 2015) were cultivated on MYAG50 and in liquid DYP (composed of DYPA without agar). Based on initial cultivation experiments, 200 mgL^−1^ chloramphenicol was added to DYPA for samples that were expected to show substantial yeast growth (fresh bee bread samples of April 2016). The antibiotic was not added to samples expected to show weak yeast growth to avoid inhibition of the already weak growth. Cultures were incubated at 30 °C for three days to one week, and cultures on solid media were performed in triplicates, except for dissected honey stomachs and pollen pellets, which were cultivated on single plates of DYPA and MYAG50 because of the small sample quantities.

### 2.3. Isolates and Identifications

Cultures were checked regularly, and yeast colonies were analysed macro- and microscopically to count according to morphotype and to select representative colonies. Representative colonies were purified, and DNA was extracted using the prepGEM Tissue kit (ZyGEM, Hamilton, New Zealand).

The D1/D2 domains of the large subunit rRNA gene (D1/D2 LSU) and, if needed, also the ITS1-5.8S-ITS2 ribosomal DNA sequences were determined after amplification using the GoTaq G2 Hot Start Colorless Master Mix (Promega, Leiden, Netherlands). The primers LR0R/LR6 were used to amplify and the primers LR0R/LR3 to sequence the D1/D2 LSU ribosomal DNA [30]. The primers ITS5/ITS4 [31] were used to amplify and sequence the ITS region. PCR was performed using an initial denaturation at 94 °C for 3 min, 30 cycles of 94 °C for 60 s, 56 °C for 90 s, 72 °C for 2 min, followed by a final extension at 72 °C for 10 min.

Partial ACT1 gene sequences were determined for representatives of the *Debaryomyces hansenii* group. The primers CA1, CA5R, CA21, and CA22R were used for amplification and sequencing [32]. PCR was performed using an initial denaturation at 94 °C for 5 min, 30 cycles at 94 °C for 30 s, at 60 °C for 30 s, 72 °C for 1 min, followed by a final extension at 72 °C for 10 min.

PCR products were sequenced by the Macrogen facility, and sequence assembly was performed in Sequencher (Gene Codes). Most similar species were determined by nucleotide BLAST searches in GenBank (https://blast.ncbi.nlm.nih.gov/Blast.cgi). Type strain sequences of those species were compared to query sequences by alignments in BioEdit 7.2.5. [33] and MEGA6 [34]. DNA sequences with any differences in comparisons to type strain sequences were deposited in the GenBank database (https://www.ncbi.nlm.nih.gov/nucleotide/) with the accession numbers MT749248-MT749273 (D1/D2 LSU), MT753010 (ITS), and MT762394, MT762395 (ACT1). Representative yeast isolates were deposited in the BCCM/MUCL culture collection (https://bccm.belspo.be/about-us/bccm-mucl) under the accession numbers MUCL 56082 to MUCL 56139, and MUCL 56143.

## 3. Results

Generally, D1/D2 LSU sequences were used to identify 252 isolates obtained from 161 samples, of which 61 samples were positive for yeast growth (Supplementary Materials Tables S1 and S2). Twenty-five species were detected (Table 2). ITS sequences distinguished closely related *Zygosaccharomyces* species. Partial ACT1 gene sequences revealed a potential new *Debaryomyces* species among isolates of the *Debaryomyces hansenii* group (Supplementary Materials Table S3). No species-level identification was effected for closely related members of the *Metschnikowia pulcherrima* clade because no barcode sequence is currently known to distinguish its members, resulting in an unresolved taxonomy [35,36]. All obtained *Metschnikowia* isolates showed unique D1/D2 LSU sequence variants (Supplementary Materials Table S4, unresolved positions marked in GenBank entries) most similar to *M. pulcherrima, Metschnikowia andauensis,* and *Metschnikowia sinensis.* They are referred to as *Metschnikowia* cf. *pulcherrima*, cf. meaning ‘compare’ from the Latin ‘confer’ to indicate that the identification is uncertain. Guided by the perception of intra-species genetic polymorphy previously reported for *Starmerella bombicola* [37,38], this name was applied to isolates that showed four to five nucleotide substitutions in the D1/D2 LSU in comparison with the type strain.

**Figure 3:**
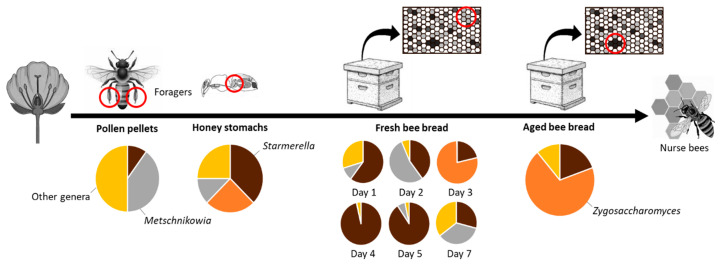
Distribution of main yeast genera associated with the gathering and storage of pollen by honey bees from pollen pellets and honey stomachs to fresh and aged been bread stored in the hives.

The most frequently detected yeast species were concentrated in three genera (Table 2, Figure 3). Pollen pellets, fresh and aged bee bread differed largely in yeast abundance and in the genera of yeasts that were isolated from them, while diverse genera were recovered from honey stomachs. *Metschnikowia* species were isolated mostly from pollen pellets, but also from honey stomachs and fresh bee bread. *Starmerella* species dominated fresh bee bread. They were also present in honey stomachs, pollen pellets, and aged bee bread. Aged bee bread was dominated by *Zygosaccharomyces* species, which were also present in fresh bee bread and honey stomachs.

**Figure 4:**
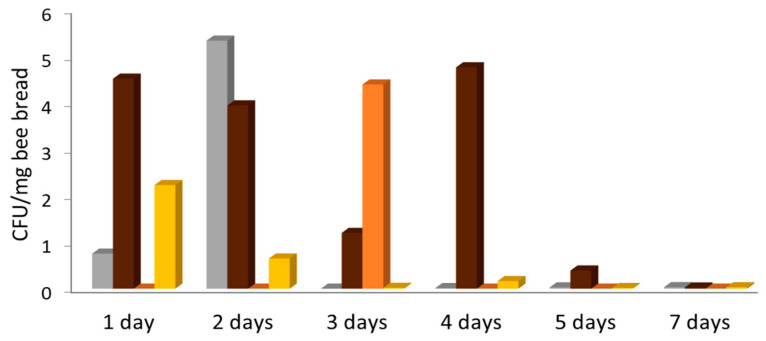
Number of yeast colonies per mg of fresh bee bread averaged over four samples at each time point. The exceptional occurrence of more than 40 CFU/mg of *S. cerevisiae* in a single seven-day sample was not included for clarity. Grey: *Metschnikowia*, brown: *Starmerella*, orange: *Zygosaccharomyces*, yellow: other genera.

All but one of 28 fresh bee bread samples showed yeast growth (Table 2). Most samples yielded two to four different yeast species. The cell density was highest with ca. 10 CFU/mg in total for all species on day 2, fluctuated during the first four days of storage, and declined towards zero on days 5 and 7 (Figure 4). One 7-day sample showed the only occurrence of *Saccharomyces cerevisiae* during this study, and this with high density. *Starmerella* (*Candida*) *apis* was the most frequent and abundant species in fresh bee bread, followed by *Starmerella magnoliae, Starmerella* (*Candida*) *apicola,* and *Starmerella bombicola*. *Metschnikowia* cf. *pulcherrima*, *Debaryomyces* spp., and *Kodamaea ohmeri* were also isolated repeatedly, but not in high abundance. *Zygosaccharomyces mellis* and *Zygosaccharomyces rouxii* were found in four samples, with high counts in two aged bee bread samples. In fresh bee bread aged one to seven days (total 754.5 mg), the average yeast count was approximately 6 CFU/mg, consisting of 3.95 CFU *Starmerella* species, 0.86 CFU *Zygosaccharomyces* species, 0.74 CFU *Metschnikowia* species, and 0.4 CFU of other yeast species (excluding *S. cerevisiae*).

Only a minority of the 107 aged bee bread samples led to the isolation of yeasts: three samples were each colonised by a single species (*Z. mellis, Zygosaccharomyces sapae, S. bombicola*), and 21 samples showed few colonies of up to two species, which belonged mostly to *Zygosaccharomyces* and rarely to *Starmerella*. The counts per mg of aged bee bread (total 1.2 g) were 1.1 CFU *Zygosaccharomyces* species and 0.9 CFU *Starmerella* species.

The most substantial counts in pollen pellets and dissected honey stomachs were owed to *Metschnikowia* (*Candida*) *rancensis* and *Metschnikowia* cf. *pulcherrima,* detected in four of the 26 samples. *Starmerella* and *Zygosaccharomyces* species typically observed in bee bread were detected in low numbers in dissected honey stomachs and rarely in pollen pellets.

Four samples of unripened honey yielded high counts of *S. bombicola* and low counts of *S. magnoliae, Z. mellis, Z. rouxii,* and *Z. siamensis*. No yeasts were obtained from surface swabs of four empty wax cells.

## 4. Discussion

The consistent isolation of yeasts from fresh bee bread and their rare isolation from aged bee bread supports the view of yeast growth in bee-stored pollen during the first days after its collection by bees [18,19]. Could the preference of bees to consume freshly stored pollen over aged bee bread [14] be linked to yeast activity? Pollen substitutes benefit from the addition of dried brewer’s yeast, a practice still in use [2], and the advantage over yeast-free substitutes has been linked to the contribution of vitamins by the yeast [39].

Yeasts of the genus *Starmerella*, detected predominantly in fresh bee bread, are well known for their close association with different bee taxa and their provisions [19,40,41,42,43,44]. They have also been documented in substrates such as flowers or sugary liquids, where they may have been vectored by insects. Through the study of bees, new *Starmerella* species have been discovered [45,46,47]. Certain *Starmerella* species appear to be associated with certain bee types, e.g., *Starmerella meliponinorum* mostly with *Tetragonista angustula,* the *S. apicola* species complex [38] with *Melipona quadrifasciata* and *Melipona rufiventris* [40], and *S. batistae* with *Diadasina distincta* and *Ptilotrix plumata* [48]. Similarily, *S.* (*Candida*) *bombi* was described and re-isolated from European bumblebee queens and associated substrates [49,50]. *Starmerella bombicola* was isolated repeatedly from bumblebee honey, though numbers varied as a function of the year and the locality [43]. A negative correlation has been established between yeasts of the genus *Starmerella* (Saccharomycetes) as the dominant part of the fungal core microbiome in pollen provisions and the bee pathogen *Ascosphaera* (Eurotiomycetes) in indoor-bred bumble bee hives, reared on sterile pollen [51].

In honey bees, *Starmerella apicola* and *S. magnoliae* were the most frequently isolated species from honey stomachs and the intestines of almost 200 pollen foragers, each species with a peak in appearance during a different season (August to September and January to June, respectively) [52]. The persistence of *S. magnoliae* in bee bread has been observed over six weeks [19].

*Starmerella apis* was the most frequently and by far the most abundantly detected yeast in the current study. This species, described from an isolate obtained from honey bee trachea in the United Kingdom [53], has been isolated with more than 20% relative abundance from individuals of *Melipona compressipes manaosensis*, but not from five other stingless bee species using non-invasive contact plate isolation in the northern savannas of Brazil [54]. *Starmerella apis* is a rare species outside the strict bee ecosystem, documented only by one survey of wine yeasts in Australia [55].

From a metabolic point of view, *Starmerella* members are nutritionally specialised, utilising only a small number of carbohydrate and nitrogen sources [56]. Several *Starmerella* species are known to produce sophorolipids in significant amounts (*S. apicola, Starmerella batistae, S. bombicola, Starmerella floricola, Starmerella gropengiesseri, Starmerella kuoi, Starmerella riodocensis, Starmerella stellata*). Its production is strain-specific and depends on culture conditions [57,58,59,60]. Sophorolipids may serve the yeast as extracellular storage material as an adaptation to high osmotic pressure caused by high sugar concentrations [61]. These compounds have attracted much interest for their surface activity and as emulsifiers due to their lipophilic and hydrophilic portions, but they also have antimicrobial activity [62,63]. Pollen grains are coated by a lipid-and-hydrocarbon-rich viscous liquid [64], and the yeasts’ sophorolipids may interact with this adhesive pollenkitt.

The repeated isolation of *Starmerella* species from bees in significant numbers suggests that their metabolic activity in the ecosystem is important. If yeasts play a role in honey bee nutrition, *Starmerella* species are the best candidates for this because they are the most frequent Apidae-associated yeasts, according to both the literature and this study [51].

The inconsistent isolation of *Zygosaccharomyces* species from a small subset of aged bee bread suggests fortuitous inoculations. Due to their high osmotolerance, these yeasts have the potential to multiply in pollen despite its low water activity. The species dominating in aged bee bread, *Z. mellis* and *Z. sapae,* are mostly known from honey and from fermentations leading to balsamic vinegar, respectively [65,66]. *Zygosaccharomyces mellis* was detected as a dominant part of the fungal community in honey-bee-collected pollen together with *Aspergillus* and *Cladosporium* using denaturing radient gel electrophoresis [67]. The infrequently obtained *Z. favi* was described as an obligate osmophile from bee bread and honey in Hungary in a study that detected a range of species similar to ours, namely *Z. rouxii, Z. mellis, Z. siamensis* [68], and *S. bombicola* [69]. Generally, *Zygosaccharomyces* species are notorious food spoilers [70] and have been reported frequently from bee-collected pollen and honey [8,40,42,43,44,47].

Our sampling of bee-collected pollen pellets and bees’ honey stomachs was intended to detect a possible transmission route of yeasts to the bee bread during transfer and storage of pollen. The detection of some bee bread yeasts in those materials allows for the possibility that *Starmerella* and *Zygosaccharomyces* enter the stored pollen either from the floral environment or from the bees’ honey stomach and were enriched in bee bread, but the low sample numbers prevent us from drawing conclusions. *Metschnikowia* cf. *pulcherrima,* detected in pollen pellets, and *Metschnikowia rancensis,* detected in pollen pellets and honey stomachs, were not the typical bee-vectored nectarivorous *Metschnikowia* species with a Palaearctic distribution, namely *M. reukaufii* and *M. gruessii* [71,72,73]. Within the large *Metschnikowia* clade, sub-clades often show a remarkable ecological and geographic specialisation and are found almost exclusively in association with floricolous beetles. The species reported in the present study belong to a sub-clade that is often encountered in fruit and associated insects. However, both species are less specialised and occur also in nectar, flowers, plant exudates, decayed wood, and nitidulid beetles [73]. *Metschnikowia* was detected as one of the most common fungal genera in honey bee guts by high-throughput sequencing [74]. The study indicated that bee-pathogen pesticides potentially alter the gut community structure, for example, by reducing the portion of Saccharomycetes versus other fungi.

Most other species encountered in this study can be regarded as generalist species, with the exceptions of *Saccharomyces cerevisiae* and *Kodamae ohmeri*. The presence of *S. cerevisiae* in one sample of fresh bee bread in high density was rather surprising because the main reservoirs of this species in nature are grape-growing environments and the guts of social wasps (*Vespa crabro*) [75]. *Saccharomyces cerevisiae* is rare in flowers and bee intestines [52,75,76,77]. The detection of *Saccharomyces* in Colony-Collapse-Disorder-affected honey bees and in young honey bees by high-throughput sequencing calls for culture-based investigation [24,25]. Our study indicates that such occurrence may be possible, although it was exceptional in honey-bee-collected pollen provisions. The ingestion of *S. cerevisiae* by bees has been linked to carbon dioxide formation in the bee’s ventriculus, followed by the death of *Nomia* bee larvae [78], and to a dysenteric condition with an elevated death rate in honey bees [79]. Interestingly, no such effects were caused by the more weakly fermenting yeast *Hanseniaspora uvarum* and an unidentified slow-growing yeast [79]. It should be noted that bees stressed by antibiotics or herbicides were prone to intestinal yeast overgrowth [80,81]. Slight *Nosema* spp. infection also lead to a strong growth of yeasts, while a heavy *Nosema* infestation resulted in a strongly lowered yeast load [82].

The yeast *K. ohmeri* can be found in diverse substrates including nitidulid beetles and flowers as their breeding and feeding sites. It is often carried by the small hive beetle (Nitidulidae: *Aethina tumida*), a facultative bee parasite. When growing on bee-collected pollen, *K. ohmeri* emits volatiles resembling honey bee alarm pheromones. The same volatiles attract additional small hive beetles to the colony, which finally becomes uninhabitable for the bees [83,84]. Our detection of *K. ohmeri* showed the presence of this component of the *A. tumida* symbiotic system, although *A. tumida* itself was not observed in the apiaries under study.

## 5. Conclusions

Freshly stored honey bee pollen in Belgium was dominated by *Starmerella* species, in particular *S. apis*. Pollen in transit by bees as pollen pellets contained *Metschnikowia* species. Extended bee bread storage favoured the presence of a dilute yeast community marked by *Zygosaccharomyces* species. The rapid decline of yeast abundance and a shift in yeast genera during the first days of pollen storage were the unique findings of this study. The apparent specialisation of different yeast genera, each in different phases of bee bread maturation or in its components, indicates a non-random distribution. More work is needed to clarify the source, potential role, and possible geographical and seasonal incidence of the yeast species that are associated with fresh bee bread to better understand honey bee nutrition. Our study suggests yeasts of the genus *Starmerella* as a study object for such work.

## Figures and Tables

**Table 1:** Cultivation media.

Name	Composition	Application
**DYPA**	Glucose 2% (Rocc, Sart-Eustache, Belgium)	General
Dextrose Yeast Extract Peptone Agar	Peptone 1% (Duchefa, Haarlem, Netherlands)	
	Yeast extract 0.5% (Oxoid, Basingstoke, United Kingdom)	
	Agar 2% (Rocc, Sart-Eustache, Belgium)	
**MA2**	Malt extract 2% (Duchefa, Haarlem, Netherlands)	General, rich in complex nutrients
Malt Agar 2%	Agar 1.5%	
**MYAG50**	Glucose 50%	Osmophiles
Malt Yeast Agar Glucose 50%	Peptone 0.5%	
	Yeast extract 0.3%	
	Malt extract 0.3%	
	Agar 2%	
**HYA**	Honey without preservatives ^1^ 3%	Basidiomycetous bee yeasts
Honey Yeast Agar [28]	Yeast extract 0.5%	
	Agar 2%	
**DG18**	Glucose 1%	Xerophilic fungi and yeasts
Dichloran-Glycerol Agar [29]	Peptone 0.5%	
	Dipotassium phosphate 0.1% (VWR, Radnor, USA)	
	Magnesium sulfate 0.05% (VWR, Radnor, USA)	
	Chloramphenicol 0.01% (AppliChem, Darmstadt, Germany)	
	Glycerol 22% (VWR, Radnor, USA)	
	Agar 1.5%	

^1^ Commercial organic honey from Mexico and Latin America.

**Table 2:** Average number of yeast colonies recovered from the specified number of bee bread and related samples.

	Fresh Bee Bread ^1^ *n* = 28		Aged Bee Bread ^2^ *n* = 107		Pollen Pellets *n* = 12		Honey Stomach *n* = 14	
	Samples	Colonies ^3^	Samples	Colonies ^3^	Samples	Colonies	Samples	Colonies
***Metschnikowia***								
*Metschnikowia (Candida) rancensis*					3	100	1	50
*Metschnikowia* cf. *pulcherrima*	9	74.4			1	10		
***Starmerella***								
*Starmerella (Candida) apicola*	10	36.5	1	0.7				
*Starmerella (Candida) apis*	18	229.8						
*Starmerella (Candida) bombi*	2	4.7	2	2			1	1
*Starmerella (Candida) magnoliae*	10	62.4	1	2	1	2	2	5
*Starmerella bombicola*	5	26.5	3	>200				
***Zygosaccharomyces***								
*Zygosaccharomyces favi*			6	3.3				
*Zygosaccharomyces mellis*	2	40.3	3	121				
*Zygosaccharomyces rouxii*	2	46	6	24.3			1	1
*Zygosaccharomyces sapae*			5	102				
*Zygosaccharomyces siamensis*	1	0.3	5	14.3			1	2
**Other**								
*Aureobasidium pullulans*	1	0.7						
*Candida parapsilosis*			1 ^4^					
*Debaryomyces hansenii*	9	5						
*Debaryomyces sp.*	2	0.7						
*Debaryomyces maramus*					2	4		
*Dothiora prunorum*	2	0.6						
*Hanseniaspora uvarum*					2	4	1	5
*Kluyveromyces dobzhanskii*					1	3	1	3
*Kodamaea ohmeri*	8	35.4						
*Meyerozyma guilliermondii*	1	0.7						
*Naganishia (Cryptococcus) diffluens*			1	1				
*Rhodotorula mucilaginosa*			2 ^4^					
*Saccharomyces cerevisiae*	1	>200						

^1^ Fresh bee bread: one to seven days, ^2^ Aged bee bread: > 20 days old. ^3^ Colony counts averaged from triplicate cultures, based on morphological types and therefore to be considered as estimates.^4^ Detected in liquid medium, no colony count performed.

## Supplementary Materials

The following are available online at https://www.mdpi.com/2076-2607/8/11/1789/s1, Table S1: Samples and their origin, weight, species, and colony counts, Table S2: Isolates, MUCL collection numbers, GenBank accession numbers, sequence similarities leading to their identification, Table S3: D1/D2 LSU sequence similarity of *Debaryomyces hansenii* group isolates, Table S4: D1/D2 LSU sequence similarity of *Metschnikowia cf. pulcherrima* isolates.
