# Supplementary material for: Specialisation of Yeast Genera in Different Phases of Bee Bread Maturation
Source: Microorganisms. 2020 Nov 14;8(11):1789. doi: 10.3390/microorganisms8111789 (PMC7696220; doi:10.3390/microorganisms8111789)
Supplement: Supplementary file 1 [file microorganisms-08-01789-s001.zip › Supplementary material.pdf]

Specialisation of yeast genera in different phases of bee bread maturation

Roxane Detry, Noa Simon-Delso, Etienne Bruneau, Heide-Marie Daniel

Supplementary Material

The following four tables are provided in a single excel file.

Table S1: Samples and their origin, weight, species, and colony counts.

In two sets of three cultures (highlighted) colony morphologies were not sufficiently discriminant and the shown colony counts are averaged over two species.

H82P3 on MYAG50: colony type c was counted on plate one 19 times, on plate two 22 times, on plate three 11 times. Two representatives of this type were identified as *S. magnoliae* and *S. apicola*. As it remains unknown how many colonies represented which of the two species, the colony number of type a were divided by two and the result assigned to the two detected species (plate one 9,5, plate two 11 and plate three 5,5 colonies).

1H82P1 on DYPA: colony type a was counted on plate 1 once, on plate 2 five times, on plate 3 twice. Two representatives of this type were identified as *S. apicola* and *D. hansenii*. As it remains unknown how many colonies represented which of the two species the colony number of type a were divided by two and the result assigned to the two detected species (plate one 0,5, plate two 2,5 and plate three 1 colonies).

Table S2: Isolates, MUCL collection numbers, GenBank accession numbers, sequence similarities leading to their identification.

Table S3: D1/D2 LSU sequence similarity of *Debaryomyces* hansenii group isolates.

Table S4: D1/D2 LSU sequence similarity of *Metschnikowia cf. pulcherrima* isolates.
